# Single Point Mutation Abolishes Water Capture in Germacradien‐4‐ol Synthase

**DOI:** 10.1002/cbic.202400290

**Published:** 2024-08-07

**Authors:** Víctor González Requena, Prabhakar L. Srivastava, David J. Miller, Rudolf K. Allemann

**Affiliations:** ^1^ School of Chemistry Main Building Cardiff University Park Place, Cardiff CF10 3AT United Kingdom

**Keywords:** Terpenoids, Natural products, Mutagenesis, Enzyme kinetics, Water capture

## Abstract

The high‐fidelity sesquiterpene cyclase (−)‐germacradien‐4‐ol synthase (GdolS) converts farnesyl diphosphate into the macrocyclic alcohol (−)‐germacradien‐4‐ol. Site‐directed mutagenesis was used to decipher the role of key residues in the water control mechanism. Replacement of Ala176, located in the G1/2 helix, with non‐polar aliphatic residues of increasing size (valine, leucine, isoleucine and methionine) resulted in the accumulation of the non‐hydroxylated products germacrene A and germacrene D. In contrast, hydroxylation was maintained when the polar residues threonine, glutamine or aspartate replaced Ala176. Additionally, although a contribution of His150 to the nucleophilic water addition could be ruled out, the imidazole ring of His150 appears to assist carbocation stabilisation. The results presented here shed light on how hydroxylating sesquiterpene synthases can be engineered to design modified sesquiterpene synthases to reduce the need for further steps in the biocatalytic production of oxygenated sesquiterpenoids.

## Graphical abstract

Engineering water capture in sesquiterpene synthase (GdolS) is explored by manipulating highly conserved active site residues. This work demonstrates that hydroxylation in sesquiterpene synthases can be prevented resulting in the formation of sesquiterpene hydrocarbons from carbocation intermediates.

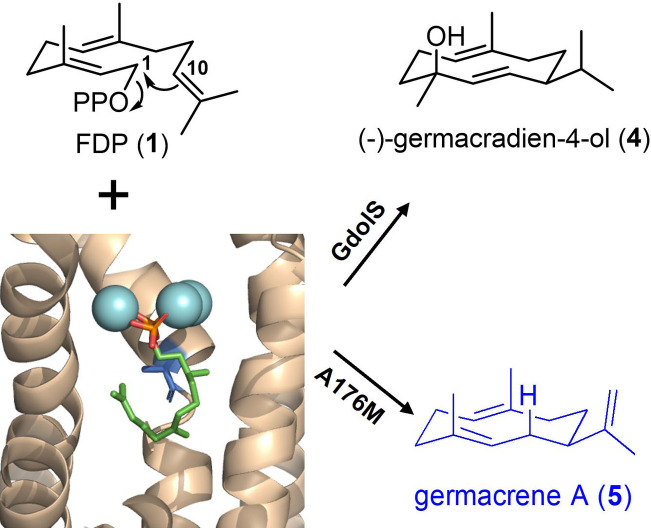

## Introduction

Terpenoids have a wide profile of essential activities in all forms of life.^[1]^ In industry, terpenoids have applications as drugs, agrochemicals, fragrances, pigments; they can serve as potential biofuels.[^2^, ^3^, ^4^, ^5^] Despite the use of a small library of acyclic diphosphate precursors, terpene synthases (TSs) have evolved to make terpenoids the largest and most structurally diverse class of natural products.^[6]^ In general, terpenoids are assembled through prenyl transferase catalysed head‐to‐tail condensations of activated isoprene units to form C_5n_ (n=1, 2, 3…) achiral isoprenyl diphosphates. Terpene synthases catalyse complex reaction cascades through high‐energy carbocationic intermediates involving intramolecular ring closures and/or hydride and alkyl shifts that are specifically quenched through proton loss or nucleophilic attack of water to generate hydrocarbon or alcohol products.[^6^, ^7^, ^8^, ^9^, ^10^] Subsequent biosynthetic processing increases the degree of functionality in terpenoid skeletons to form oxidised, methylated, dehydrogenated and acetylated compounds.[^11^, ^12^, ^13^] A small number of terpene synthases can generate hydroxylated products through regio‐ and stereoselective ‘water capture’, which represents a little‐explored biological pathway to oxygen‐containing terpenes.[^14^, ^15^, ^16^, ^17^, ^18^, ^19^, ^20^] Thus, the organisation of active site water molecules is a key element for product selectivity in terpene synthases catalysis.[^21^, ^22^]

The diversity in terpene structure and stereochemistry is precisely determined by the binding and folding of the acyclic diphosphate substrate in the active site of terpene synthases.[^23^, ^24^, ^25^, ^26^] Class I terpene synthases share an α‐helical bundle, containing up to three domains.^[6]^ In the catalytic domain, the conserved DDxxD/E and NSE/DTE motifs face one another across the active site entrance and guide the substrate through Mg^2+^‐diphosphate (PP_i_)‐enzyme cluster formation.[^25^, ^27^, ^28^] This event triggers active site closure and substrate ionisation; subsequent intermediate carbocations are sheltered from premature solvent quench.[^19^, ^25^, ^29^, ^30^] Additionally, the hydrophobic active site composition allows carbocation stabilisation through cation‐π interactions *via* aromatic side chains.[^6^, ^31^, ^32^, ^33^] Aliphatic residues are involved in forming the active site template, creating an ensemble governing substrate and intermediate conformation.[^34^, ^35^]

X‐ray crystal structures of terpenoid synthases frequently show water molecules trapped in the active site.[^19^, ^22^, ^36^] Terpene synthases possess a sophisticated architecture in which water management is crucial, but information on this strategy is sparse. These water molecules may form part of the active site template for correct product formation or to perform an active role in catalysis by quenching a carbocation to generate hydroxylated products.[^22^, ^37^, ^38^, ^39^] However, aberrant water molecules may limit the catalytic performance.^[40]^

Previously, to gain structural insights into water‐capture mechanisms, closed enzyme‐ligand complexes in aristolochene synthase from *Aspergillus terreus* (ATAS) were examined. Mutagenesis studies of active site water‐binding residues (Q151, N299 and S203) revealed that even though ATAS does not generate hydroxylated products, water is part of the substrate/intermediates template contour and plays a silent role in controlling reaction fidelity.^[22]^ In δ‐cadinene synthase (DCS), the active site has been engineered to produce (−)‐germacradien‐4‐ol, altering natural discrimination between deprotonation and water capture.[^41^, ^42^] In copalyl diphosphate/*ent*‐kaurene synthase, a single amino acid substitution abolishes the addition of a water molecule.^[43]^ Recent combined computational and experimental work in our group identified two regions of TSs, namely the G_helix_ and a RQH motif, which hold water molecules and are involved in hydroxylation of neutral intermediates.^[44]^ However, understanding the precise mechanism for such changes remains a challenge. Water‐capture in TSs is tunable and further delineation of the structural requirements for the control of water molecules in the active site will have a significant impact on the engineering of bespoke terpenoid synthases to produce novel terpenoids with diverse applications.

**Scheme 1:**
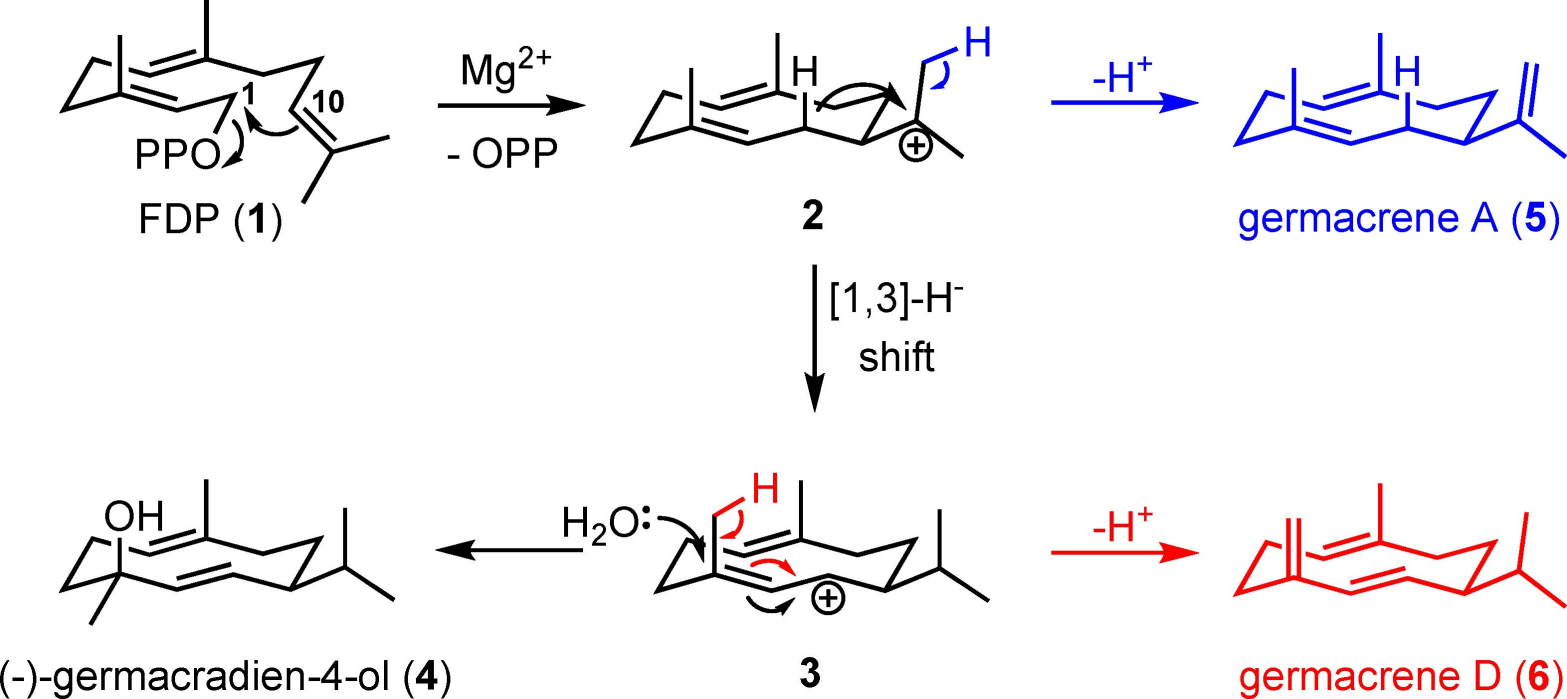
Catalytic mechanisms for the GdolS (black) and mutant (blue and red) catalysed conversions of FDP (**1**) to (−)‐germacradien‐4‐ol (**4**), germacrene A (**5**), and germacrene D (**6**) via intermediates **2** and **3**.

The bacterial sesquiterpene synthase (−)‐germacradien‐4‐ol synthase (GdolS) from *Streptomyces citricolor* catalyses the Mg^2+^ dependent formation of (−)‐germacradien‐4‐ol (**4**) from farnesyl diphosphate (**1**, FDP, Scheme 1). GdolS acts with high fidelity and has a relatively simple catalytic mechanism; it is therefore a good model to examine active site water control in terpene synthases. The catalytic mechanism of GdolS is proposed to proceed through Mg^2+^‐mediated PP_i_ cleavage followed by 1,10‐ring closure to form (*E*,*E*)‐germacradienyl tertiary carbocation (**2**).^[36]^ GdolS then facilitates 1,3‐hydride shift to generate the germacradienyl allyl carbocation **3**, protecting intermediate **2** from proton loss or water addition. Lastly, GdolS allows selective water addition to **3** to form (−)‐germacradien‐4‐ol (**4**), avoiding proton loss and formation of germacrene D (**6**) (Scheme 1).[^16^, ^36^]

Previous mutagenesis had revealed that GdolS‐N218Q generates similar amounts of germacradien‐4‐ol (**4**) and germacrene A (**5**), but the catalytic activity was reduced relative to the GdolS.^[36]^ N218 is part of the NSE motif and the drastic reduction in catalytic efficiency may have arisen from disturbing the enzyme conformation around the Mg^2+^_3_ cluster. No suitable water binding residues were identified in the active site and it was postulated that loop movements may facilitate entry of bulk water to the active site.^[36]^ To date, GdolS has only been crystallised in its open apo form.^[36]^ The absence of a closed crystal structure in complex with a ligand restricts interpretation of the enzymatic water governance in GdolS. In the closed structure, substrate binding promotes conformational changes, namely translation and rotation of α‐helices C, D, G, H and K towards the active site cavity to accurately position the acyclic precursor for initial cyclisation and assist in carbocation stabilisation and final product formation. Computational studies and homology modelling have previously shed light on the mechanistic details in terpenoid synthase catalysis.[^25^, ^45^, ^46^, ^47^, ^48^, ^49^] Hence, the active site of GdolS was re‐explored with the assistance of the available closed X‐ray crystal structures of related sesquiterpene synthases.

This investigation aimed to convert GdolS into a non‐hydroxylating enzyme. Three alternative approaches were considered for abolishing the water capture catalysed by GdolS; 1) water can be part of a hydrogen bonding network, that could be compromised by altering the active site; 2) strategic location of polar amino acids to quench the final carbocation; and 3) water can enter through a channel at the right time and this could be disrupted by altering enzyme conformational ensembles.

**Figure 1:**
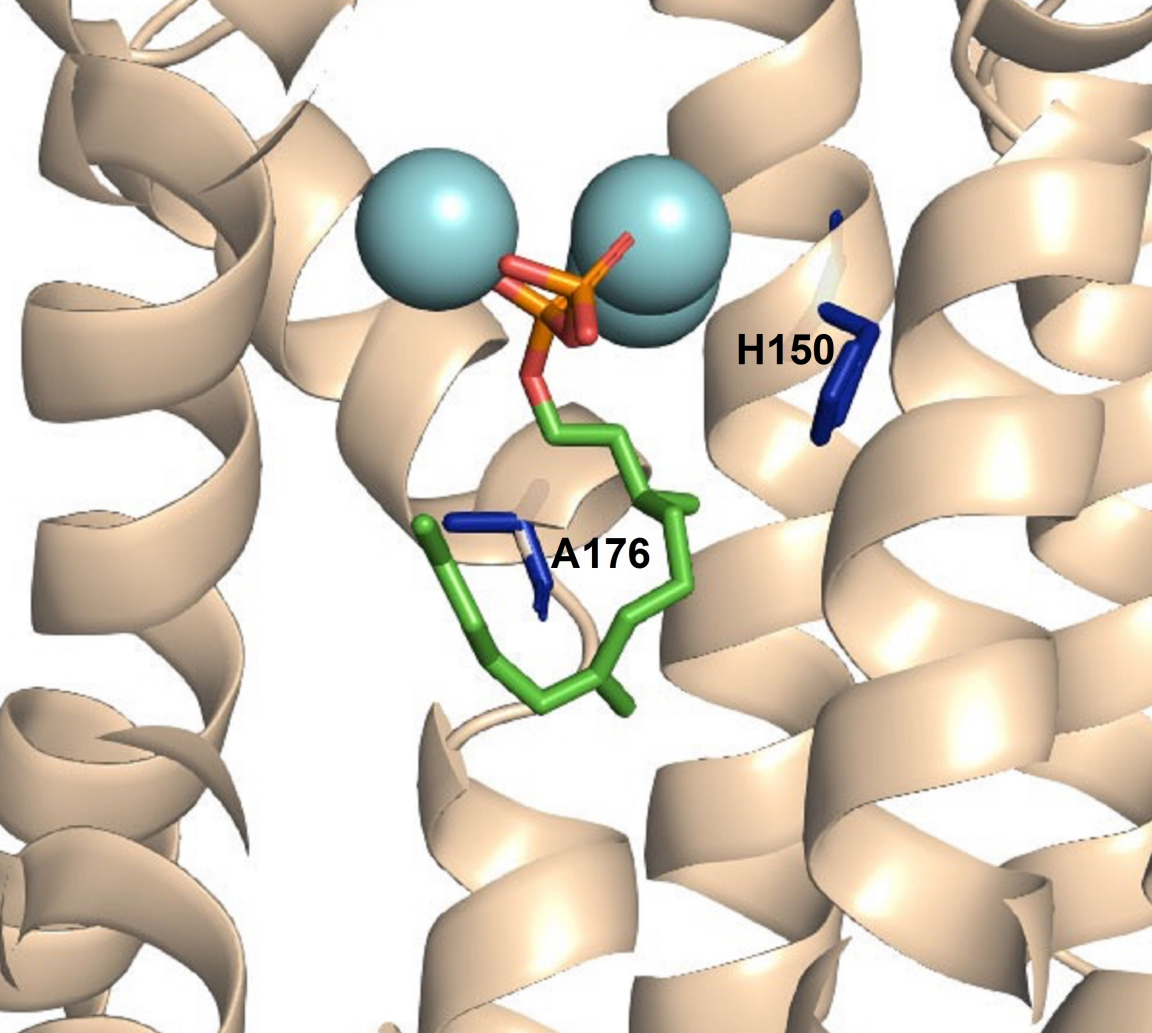
Cartoon representation of the X‐ray crystal structure of GdolS^[36]^ in complex with 2,3‐dihydrofarnesyl diphosphate, docked from SdS (4KOM).^[19]^ Amino acids H150 and A176 are highlighted in dark blue.

**Figure 2:**
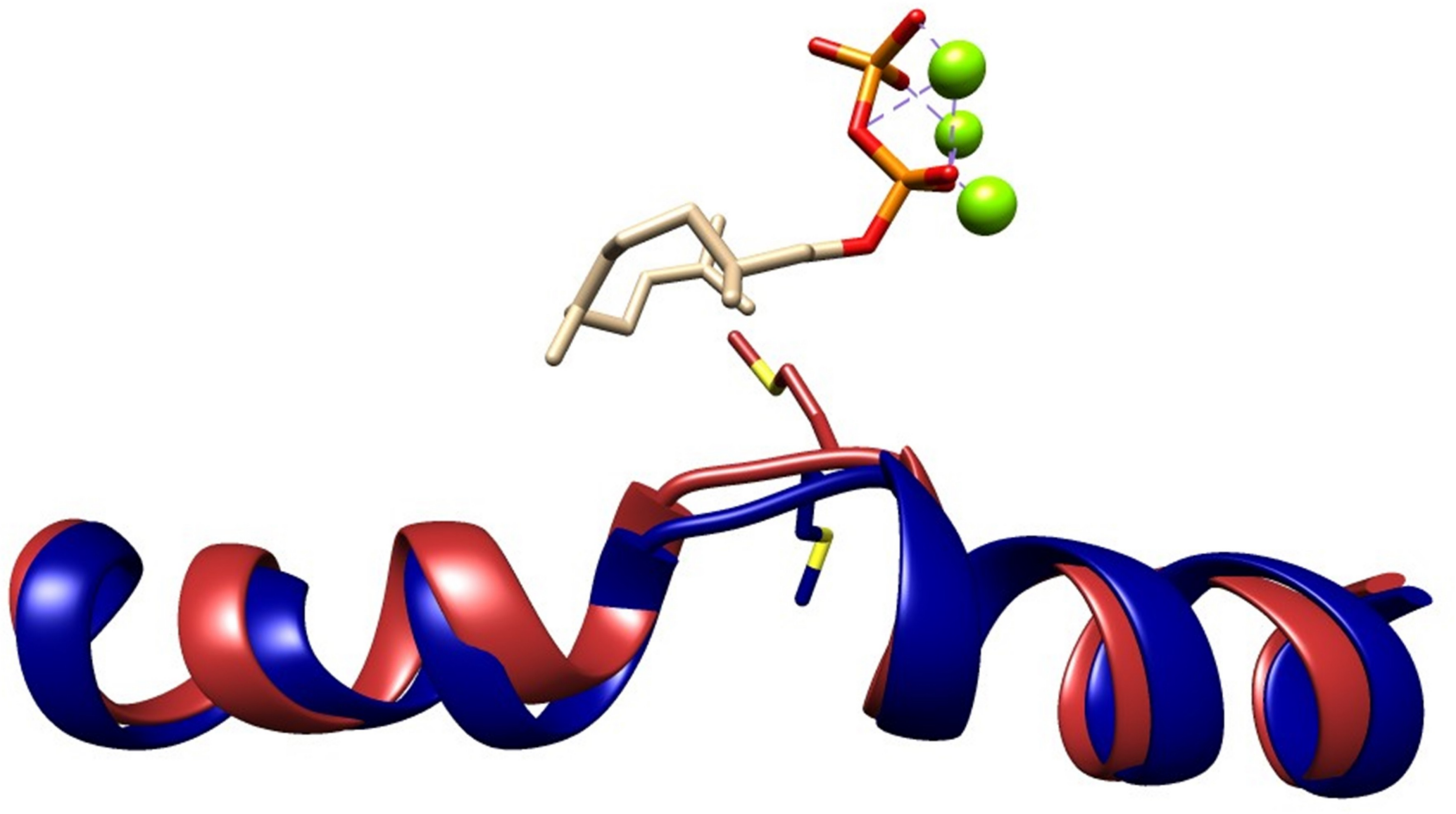
Cartoon representation showing the superposition of 2,3‐dihydrofarnesyl diphosphate complexes of GdolS‐A176M, built using GdolS (5I1U)^[36]^ as template (blue), and GdolS‐A176M built using SdS (4KOZ)^[19]^ as template (red).

A G1/2 helix break motif, widely conserved in class 1 terpene synthases, has been shown to play an important structural role for catalysis in *inter alia* hedycaryol synthase (HcS),^[39]^ δ‐cadinene synthase (DCS)^[41]^ and 1,8‐cineole synthase (Sf‐CinS1).^[50]^ In selinadiene synthase (SdS) from *Streptomyces prestinaespiralis*, residues R172, D181 and G182 form a catalytic triad called diphosphate sensor, linker and effector motif, which is proposed to assist in substrate recognition, active‐site closure, substrate ionisation and product distribution.^[19]^ The overall composition and orientation of this helix break motif is highly conserved among bacterial terpenoid synthases.[^19^, ^39^, ^41^, ^42^] The carbonyl oxygen of G182, a residue within this kink region of SdS, was recently shown together with a water molecule as both a base and an acid during catalysis, and hence it plays a crucial role in product distribution.^[51]^ In GdolS, this catalytic triad is made up of R172, T175 and A176. The catalytic role of R172 in diphosphate binding was previously analysed where replacement with lysine (R172K) resulted in similar profile to GdolS along with minor proportions of **5** and **6** with significant decrease in catalytic efficiency. However, replacement of R172 with glutamate resulted in an inactive variant.^[52]^ The function of T175 and specially A176 in GdolS, have not been investigated (Figure 1). Herein, site‐directed mutagenesis was employed to elucidate the role of A176 in GdolS catalysis, which is proximal to the substrate and a potential contributor to loop movements that may open and close a water channel through the active site. The positioning of A176M in the model generated using apo and closed templates reflects on the structural changes of G1/2 helix break upon substrate binding (Figure 2). Additionally, based on previous investigations of ATAS catalysis, the role of H150 in water activation by GdolS was examined (Figure 1). The results show that the A176 side chain is essential for catalysis and product fidelity. Single A176I and A176M mutations significantly alter product distributions, switching GdolS into functional germacrene A synthases.

## Results and Discussion

Here we present the analysis by gas chromatography‐mass spectrometry (GC‐MS) of the pentane‐extractable products generated from incubations of 17 GdolS mutants with FDP (**1**). Kinetic constants were determined for all the variants.

### G1/2 Helix Break Amino Acids

**Figure 3:**
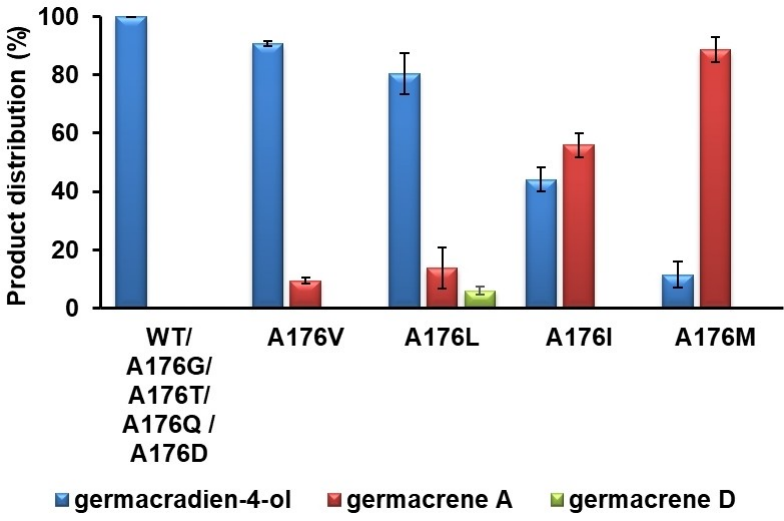
Bar chart of the organic extractable product distribution (%) generated by GdolS and GdolS‐A176 mutants.

To examine the role of A176 in GdolS catalysis, alanine was replaced by hydrophobic and hydrophilic residues of varying size (Figure 3). In general, substitution of non‐polar amino acids for A176 led to different product profiles, whereas replacement of polar amino acids for A176 did not affect product distribution. Firstly, A176 was replaced by glycine, a more flexible amino acid with reduced size. The product distribution was not altered in comparison to GdolS. In contrast, the replacement of A176 with valine, which has roughly twice the van der Waals volume of alanine, resulted in a slightly shifted product distribution, producing 91 % germacradien‐4‐ol (**4**) and 9 % germacrene‐A (**5**) in the organic extractable products (Figure 3). Aliphatic residues have been shown to act in concert with aromatic counterparts to force **1** into the correct productive conformation for cyclisation.^[34]^ In this case, the absence of acyclic (farnesene) products shows that replacement of the methyl group for a bulkier aliphatic side chain does not sterically affect the cyclisation reaction in GdolS, ruling out any contribution of A176 to positioning and orientation of the C10−C11 double bond prior to cyclisation. GdolS‐A176T and GdolS‐A176L provided more information about the steric and electrostatic contribution of A176 to catalysis. Incubation of GdolS‐A176L with **1** led to the accumulation of germacrene A (**5)** (14 %) and germacrene D (**6**, 6 %), products that arise from premature deprotonation of intermediates **2** and **3** respectively (Scheme 1) along with **4** (80 %). On the other hand, GdolS‐A176T displayed a product profile like that of GdolS. The WT‐like product profile upon substitution of A176 for a larger but polar side chain seems to rule out water activation as the main role for A176, as otherwise a steric displacement of an activated water molecule and formation of aberrant products would be expected, as observed for GdolS‐A176V and GdolS‐A176L (non‐polar switches).^[43]^ Instead, A176 may play a more passive catalytic role as a part of the template contour. In terpene synthases, X‐ray crystal structures often show hydrogen bonding networks in the active site, in which water interacts with the diphosphate motif, Mg^2+^ ions and/or polar side chains.^[22]^ Also, A176 could be close to an aromatic side chain responsible for a cation‐π interaction that stabilizes intermediate **3**.[^6^, ^31^, ^32^] Larger aliphatic groups can disrupt these associations and prompt a structural adjustment that results in an alternate template and hence favouring the deprotonation of carbocation intermediates **2** and **3** to produce **5** and **6** instead of water attack on intermediate **3**. To probe this hypothesis, A176 was replaced by isoleucine, methionine, phenylalanine, glutamine and aspartate, respectively. GdolS‐A176F was inactive, most likely due to perturbation of the active site. The isoleucine variant generated 44 % and 56 % of **4** and **5**, respectively. Although leucine and isoleucine have similar van der Waals volumes their different conformations influence the reaction termination unequally, highlighting the delicate intrinsic structural role of A176. Consequently, GdolS‐A176M exhibits a significant change in product distribution with the formation of a 9 : 1 mixture of **5** and **4** as the predominant reaction products. Moreover, for GdolS‐A176Q and A176D, formation of only **4** was observed upon incubation with **1** (Figure 3).

To assess their catalytic competence kinetic parameters (*k*_cat_ and *K*_M_) were measured for the GdolS‐A176 mutants (Table 1). In general, *K*_M_ values for all mutants are similar to those measured for GdolS.^[36]^ The most significant difference is observed in GdolS‐A176Q, which is 40‐fold higher than that for the GdolS and 20‐fold decrease in *k*_cat_ resulting in substantial change in catalytic efficiency. The charge distribution in the active site could significantly affect substrate binding and ionisation. However, GdolS‐A176G, GdolS‐A176V and GdolS‐A176L display values for *k*_cat_ about 10‐fold lower than those of the wildtype enzyme (GdolS). Whereas GdolS‐A176M and GdolS‐A176D show an approximately 100‐fold decrease in *k*_cat_ relative to GdolS (Table 1). The decrease in catalytic efficiency is mainly due to reduction in *k*_cat_ (Table 1). These findings suggests that the volume of the side chain of residue‐176 plays an important role in catalysis.

**Table 1:** Kinetic parameters of GdolS and GdolS‐mutants.

Enzyme	*k*_cat_ [×10^−3^ s^−1^]	*K*_M_ [μM]	*k*_cat_/*K*_M_ [×10^3^ M^−1^ s^−1^]
GdolS^[36]^	79±3	1.07±0.13	73.8±9.4
GdolS‐T175D	n/a^[a]^	n/a^[a]^	n/a^[a]^
GdolS‐T175C	5±0.12	0.83±0.08	6.03±0.63
GdolS‐T175N	3±0.09	1.01±0.10	2.97±0.31
GdolS‐A176G	10±0.3	1.61±0.18	6.2±0.7
GdolS‐A176V	8±0.2	1.31±0.12	6.12±0.60
GdolS‐A176L	13±0.6	3.70±0.43	3.51±0.44
GdolS‐A176I	6±0.3	1.39±0.19	4.32±0.63
GdolS‐A176M	0.83±0.07	3.03±0.66	0.27±0.06
GdolS‐A176F	n/a^[a]^	n/a^[a]^	n/a^[a]^
GdolS‐A176D	0.9±0.05	2.41±0.35	0.37±0.06
GdolS‐A176T	2±0.03	0.67±0.05	2.97±0.22
GdolS‐A176Q	4±0.94	44.83±14.15	0.09±0.04
GdolS‐H150Y	11±0.36	1.91±0.20	5.77±0.64
GdolS‐H150F	1±0.04	1.81±0.21	0.55±0.68
GdolSH150W	n/m^[b]^	n/m^[b]^	n/m^[b]^
GdolS‐H150C	n/m^[b]^	n/m^[b]^	n/m^[b]^
GdolS‐H150R	n/m^[b]^	n/m^[b]^	n/m^[b]^

[a] Not applicable: inactive enzymes. [b] No measurable activity; activity too low for kinetic parameters to be determined.

In SdS,^[19]^ D181 (T175 in GdolS) in the G‐helix shifts upon FDP binding to interact with the guanidinium group of R178 (R172 in GdolS) and triggers a structural rearrangement of G182 (A176 in GdolS). GdolS‐T175C and GdolS‐T175N were able to functionally substitute for threonine in the hydrogen‐bonding network, with no change in product distribution albeit having lower *k*_cat_ values. In contrast, GdolS‐T175D was inactive.

### Water Binding Residues

**Figure 4:**
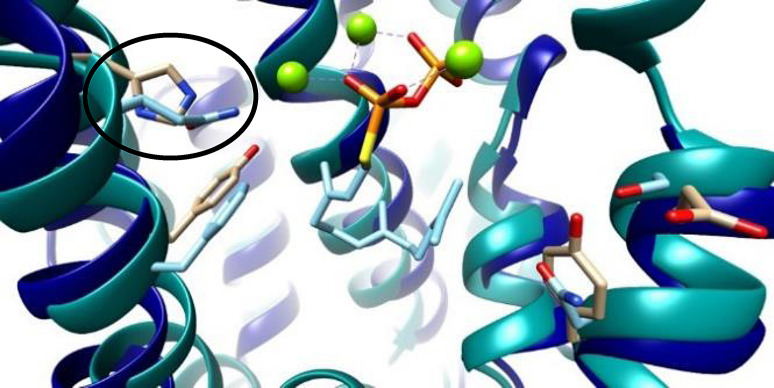
Cartoon representation of the X‐Ray co‐crystal structures of GdolS^[36]^ (blue) and ATAS (green)^[53]^ in complex with farnesyl S‐thiolodiphosphate. Highlighted in black circle H150 in GdolS/Q151 in ATAS.

Previous experiments with GdolS highlighted Y303 and E307 as potential water binding residues.^[36]^ These side chains were substituted for amino acids with or without hydrogen bonding capabilities, which resulted in little or no change in the water capture behaviour, thus ruling out the possibility of their involvement in water activation.^[36]^ In ATAS,^[22]^ two trapped water molecules were shown to bond with the Q151 side chain in the upper active site, and the alteration of these interactions led to the generation of hydroxylated products. Sequence alignment and crystal structure superposition of AT‐AS with GdolS (4KUX and 5I1U, respectively) showed that this residue corresponds to H150 in GdolS (Figure 4). Hence H150 was replaced with Y, F, W, C and R, respectively. GdolS‐H150Y and GdolS‐H150C were generated to test the possibility that water binding could be restored with these alternative polar functional groups, abolishing water capture and to test the influence of the aromatic chain in GdolS catalysis. Both GdolS‐H150Y and GdolS‐H150C are catalytically active, and the only organic product was **4**. These results rule out water activation as a possible role for H150. Interestingly, GdolS‐H150C was of much diminished catalytic activity, while GdolS‐H150Y was only around 10‐times less efficient than the wild‐type enzyme (GdolS). This implies that H150 may govern cation‐π stabilization of an intermediate carbocation. To test this, GdolS‐H150R was generated but this enzyme showed no measurable catalytic activity. GdolS‐H150F and GdolS‐H150W were also created. Both enzymes were functional GdolSs, but GdolS‐H150W does not show measurable catalytic activity (Table 1), most likely because of steric effects due to the large size of the indole ring.

## Conclusions

In this work, GdolS has been engineered to produce various non‐hydroxylating enzymes. A176, located in the G1/2 helix break, plays a key structural role in governing the reaction pathway. Non‐polar aliphatic residues modulate the substrate template contour and water capture or deprotonation of the final carbocationic reaction intermediate. Replacement of alanine 176 with glycine had no impact on the product profile. However, when A176 was replaced with larger non‐polar aliphatic residues (valine, leucine, isoleucine and methionine) the outcome switched to the production of germacrene‐A (**5**) along with germacradien‐4‐ol (**4**) (Figure 3) with **5** being the major product for A176M. The lack of acyclic products reflects that alanine 176 is crucial in the later part of the reaction (Scheme 1), which is also supported with kinetic studies showing similar *K*_M_ values for these variants with only minor variations in *k*_cat_. The G1/2 helix break has been postulated as a key region for water access through a channel in other sesquiterpene synthases.[^42^, ^44^] To judge whether this is a consequence of a steric energy barrier to water influx, A176 was also replaced with polar amino acids of varying sizes. Indeed, substitutions A176D, A176T and A176Q resulted in functional GdolS and ruled out this hypothesis. The combination of these results suggest that A176 might be in a place near an active site water molecule, which could form part of a hydrogen‐bonding network. The ‘spectator’ role of alanine 176 is compromised upon substitution with larger non‐polar amino acids that break the hydrogen‐bonding network and hence result in aberrant proton loss from the intermediates germacradienyl tertiary carbocation (**2**) and germacradienyl allyl carbocation (**3**). Furthermore, these associations (hydrogen‐bonding network) can be restored by polar amino acid side chains such as threonine, glutamine and aspartate.

The broad application of natural terpenoids in industry often relies on access to functionalised hydrocarbons such as oxidised terpenes. Engineering water capture in terpene synthases may provide a cost‐effective approach to produce oxygen containing terpenes and reduce the need to use P450 enzymes or complex and often costly non‐enzymatic chemical transformations.

## Experimental Section

All chemicals were acquired from Sigma‐Aldrich, Fisher or Melford. except radiolabeled substrate [1‐^3^H‐FDP], which was purchased from Fluorochem. Protein purification and characterisation, tables of mutagenic primers, gas chromatograms from enzymatic incubations, mass spectra, and kinetic data are described in the electronic supplementary information.

### Materials and Methods

***Preparation of GdolS (and mutants)***. The expression vector containing the gene encoding GdolS (SC1) from *Streptomyces citricolor* was a generous gift from Y. Onishi (University of Tokyo). pET16b‐GdolS contains an in‐frame *N*‐decahistidine. pET‐GdolS was introduced into BL21(DE3) chemically competent cells and positive colonies were selected on an LB‐agar plate containing 100 μg mL^−1^ ampicillin. A single transformed colony was used to inoculate 100 mL of LB medium containing antibiotic (100 μg mL^−1^ ampicillin). The culture was grown at 37 °C overnight whilst shaking (150 rpm). 5 mL of this culture was transferred to 500 mL of LB medium containing ampicillin (100 μg mL^−1^). The culture was incubated at 37 °C until OD_600_=0.6 was reached and was then induced with β‐D‐1‐thiogalactopyranoside (IPTG, 0.2 mM) and allowed to grow for an additional 3 h. Cells were harvested by centrifugation at 4 °C (3400 *g*, 10 min) and stored in −20 °C until further use. The pellet was thawed on ice and resuspended in cell lysis buffer (40 mL, 50 mM Tris, 100 mM NaCl and 10 mM imidazole, pH 8). Cells were disrupted by sonication (4 °C, 5 min, pulse 5 s on/10 s off, 40 % amplitude) and the resulting suspension was centrifuged at 18000 *g* for 30 mins at 4 °C. SDS‐PAGE showed that protein of expected size (~35 kDa) was present in the supernatant solution, and this was applied to a Ni‐NTA affinity column (Expedeon, 5 cm) preequilibrated with lysis buffer. The column was washed with a gradient of imidazole (from 10 mM to 500 mM in lysis buffer, over 10 column volumes), and the fractions were analysed by SDS‐PAGE. The fractions containing >90 % pure GdolS (molecular weight 38,687) were combined, dialysed for 16 h (10 mM Tris, pH 8) using MWCO 12–14 kDa membrane and then concentrated to 10 mL using Amicon YM 30. The protein concentration was measured using the method of Bradford^[54]^ and aliquots stored at 0 °C.

Site‐directed mutagenesis (SDM) technique is described in the Supporting information and pairs of mutagenic primers are given (Section 2). The expression and purification of GdolS mutant enzymes was carried out as described for GdolS.

***Analytical incubations of GdolS and mutants with FDP (1)***. FDP (200 μM) was added to incubation buffer (250 μL; 50 mM Tris, 5 mM MgCl_2_, 5 mM βME, pH 8) followed by addition of enzyme solution (1 μM). The reaction mixture was overlaid with 1 mL pentane and incubated at room temperature for 6 h with gentle shaking. Organic soluble products were extracted into the pentane layer by vortexing the mixture for 30 s and analysed by GC‐MS (See the supporting information, Sections 1 and 4). Negative controls (no enzyme) were also performed to ensure that products analysed arose from enzyme catalysis.

***Steady‐state kinetics of GdolS‐mutants***. Kinetic assays were performed according to the standard, linear range, micro‐assay procedure previously developed for GdolS (supporting information, Section 5).

***Homology modelling of GdolS and mutants***. Homology models were conducted using the SWISS‐MODEL workspace,^[45]^ based on the crystal structure of germacradien‐4‐ol (GdolS, 5I1U), selina‐4(15),7(11)‐diene synthase (SdS, 4OKZ), *Aspergillus terreus* aristolochene synthase (ATAS, 4KUX and 4KWD) and *P. roqueforti* aristolochene synthase (PRAS, 1F1P).

## Supporting Information Summary

The details of materials and general methods, mutagenesis primers, PCR conditions, GC chromatograms and kinetics are included in the Supporting Information.

## Conflict of Interests

The authors declare no conflict of interest.

1

## Supporting information

As a service to our authors and readers, this journal provides supporting information supplied by the authors. Such materials are peer reviewed and may be re‐organized for online delivery, but are not copy‐edited or typeset. Technical support issues arising from supporting information (other than missing files) should be addressed to the authors.

Supporting Information

## Data Availability

Raw data for GC‐MS chromatograms and kinetic characterisation of GdolS and mutants are available at https://doi.org/10.17035/d.2024.0304658740.

## References

[1] J. Gershenzon , N. Dudareva , Nat. Chem. Biol. 2007, 3, 408–414. 17576428 10.1038/nchembio.2007.5

[2] A. J. Hick , M. C. Luszniak , J. A. Pickett , Nat. Prod. Rep. 1999, 16, 39–54.

[3] S. K. Lee , H. Chou , T. S. Ham , T. S. Lee , J. D. Keasling , Curr. Opin. Biotechnol. 2008, 19, 556–563. 18996194 10.1016/j.copbio.2008.10.014

[4] M. E. Maffei , J. Gertsch , G. Appendino , Nat. Prod. Rep. 2011, 28, 1359–1380. 21670801 10.1039/c1np00021g

[5] P. P. Peralta-Yahya , M. Ouellet , R. Chan , A. Mukhopadhyay , J. D. Keasling , T. S. Lee , Nat. Commun. 2011, 2, 483. 21952217 10.1038/ncomms1494 PMC3195254

[6] D. W. Christianson , Chem. Rev. 2017, 117, 11570–11648. 28841019 10.1021/acs.chemrev.7b00287 PMC5599884

[7] D. J. Miller , R. K. Allemann , Nat. Prod. Rep. 2012, 29, 60–71. 22068697 10.1039/c1np00060h

[8] J. S. Dickschat , Nat. Prod. Rep. 2016, 33, 87–110. 26563452 10.1039/c5np00102a

[9] M. B. Quin , C. M. Flynn , C. Schmidt-Dannert , Nat. Prod. Rep. 2014, 31, 1449–1473. 25171145 10.1039/c4np00075g PMC4167380

[10] D. E. Cane , H. Ikeda , Acc. Chem. Res. 2012, 45, 463–472. 22039990 10.1021/ar200198d PMC3288161

[11] E. Pichersky , J. P. Noel , N. Dudareva , Science 2006, 311, 808–811. 16469917 10.1126/science.1118510 PMC2861909

[12] D. K. Ro , E. M. Paradise , M. Quellet , K. J. Fisher , K. L. Newman , J. M. Ndungu , K. A. Ho , R. A. Eachus , T. S. Ham , J. Kirby , M. C. Y. Chang , S. T. Withers , Y. Shiba , R. Sarpong , J. D. Keasling , Nature 2006, 440, 940–943. 16612385 10.1038/nature04640

[13] B. Zhao , X. Lin , L. Lei , D. C. Lamb , S. L. Kelly , M. R. Waterman , D. E. Cane , J. Biol. Chem. 2008, 283, 8183–8189. 18234666 10.1074/jbc.M710421200 PMC2276382

[14] W. K. W. Chou , I. Fanizza , T. Uchiyama , M. Komatsu , H. Ikeda , D. E. Cane , J. Am. Chem. Soc. 2010, 132, 8850–8851. 20536237 10.1021/ja103087w PMC2904616

[15] D. E. Cane , M. Tandon , P. C. Prabhakaran , J. Am. Chem. Soc. 1993, 115, 8103–8106.

[16] C. Nakano , F. Kudo , T. Eguchi , Y. Ohnishi , ChemBioChem 2011, 12, 2271–2275. 23106076 10.1002/cbic.201100418

[17] C. Nakano , S. Horinouchi , Y. Ohnishi , J. Biol. Chem. 2011, 286, 27980–27987. 21693706 10.1074/jbc.M111.265652 PMC3151043

[18] C. M. Wang , R. Hopson , X. Lin , D. E. Cane , J. Am. Chem. Soc. 2009, 131, 8360–8361. 19476353 10.1021/ja9021649 PMC2702122

[19] P. Baer , P. Rabe , K. Fischer , C. A. Citron , T. A. Klapschinski , M. Groll , J. S. Dickschat , Angew. Chem. Int. Ed. 2014, 53, 7652–7656. 10.1002/anie.201403648 24890698

[20] D. E. Cane , R. M. Watt , Proc. Natl. Acad. Sci. U. S. A. 2003, 100, 1547–1551. 12556563 10.1073/pnas.0337625100 PMC149869

[21] S. C. Hammer , P. O. Syrén , B. Hauer , ChemistrySelect 2016, 1, 3589–3593.

[22] M. Chen , W. K. W. Chou , N. Al-Lami , J. A. Faraldos , R. K. Allemann , D. E. Cane , D. W. Christianson , Biochemistry 2016, 55, 2864–2874. 27172425 10.1021/acs.biochem.6b00343 PMC4879067

[23] M. J. Calvert , P. R. Ashton , R. K. Allemann , J. Am. Chem. Soc. 2002, 124, 11636–11641. 12296728 10.1021/ja020762p

[24] P. N. Blank , G. H. Barrow , W. K. W. Chou , L. Duan , D. E. Cane , D. W. Christianson , Biochemistry 2017, 56, 5798–5811. 28967743 10.1021/acs.biochem.7b00895 PMC5664225

[25] M. W. Van der Kamp , J. Sirirak , J. Zurek , R. K. Allemann , A. J. Mulholland , Biochemistry 2013, 52, 8094–8105. 24106830 10.1021/bi400898k

[26] R. Li , W. K. W. Chou , J. A. Himmelberger , K. M. Litwin , G. G. Harris , D. E. Cane , D. W. Christianson , Biochemistry 2014, 53, 1155–1168. 24517311 10.1021/bi401643u PMC3985761

[27] E. Y. Shishova , L. Di Costanzo , D. E. Cane , D. W. Christianson , Biochemistry 2007, 46, 1941–1951. 17261032 10.1021/bi0622524 PMC2518937

[28] J. A. Aaron , D. W. Christianson , Pure Appl. Chem. 2010, 82, 1585–1597. 21562622 10.1351/PAC-CON-09-09-37 PMC3090183

[29] J. A. Aaron , X. Lin , D. E. Cane , D. W. Christianson , Biochemistry 2010, 49, 1787–1797. 20131801 10.1021/bi902088z PMC2840623

[30] E. Y. Shishova , F. Yu , D. J. Miller , J. A. Faraldos , Y. Zhao , R. M. Coates , R. K. Allemann , D. E. Cane , D. W. Christianson , J. Biol. Chem. 2008, 283, 15431–15439. 18385128 10.1074/jbc.M800659200 PMC2397452

[31] D. A. Dougherty , Science 1996, 271, 163–168. 8539615 10.1126/science.271.5246.163

[32] J. A. Faraldos , A. K. Antonczak , V. González , R. Fullerton , E. M. Tippmann , R. K. Allemann , J. Am. Chem. Soc. 2011, 133, 13906–13909. 21815676 10.1021/ja205927u

[33] S. Das , M. Dixit , D. T. Major , Bioorg. Med. Chem. 2016, 24, 4867–4870. 27427398 10.1016/j.bmc.2016.07.002

[34] J. A. Faraldos , V. González , M. Senske , R. K. Allemann , Org. Biomol. Chem. 2011, 9, 6920–6923. 21870004 10.1039/c1ob06184d

[35] Z. Li , R. Gao , Q. Hao , H. Zhao , L. Cheng , F. He , L. Liu , X. Liu , W. K. W. Chou , H. Zhu , D. E. Cane , Biochemistry 2016, 55, 6599–6604. 27933789 10.1021/acs.biochem.6b01004 PMC5154624

[36] D. J. Grundy , M. Chen , V. González , S. Leoni , D. J. Miller , D. W. Christianson , R. K. Allemann , Biochemistry 2016, 55, 2112–2121. 26998816 10.1021/acs.biochem.6b00115 PMC4829482

[37] D. A. Whittington , M. L. Wise , M. Urbansky , R. M. Coates , R. B. Croteau , D. W. Christianson , Proc. Natl. Acad. Sci. U. S. A. 2002, 99, 15375–15380. 12432096 10.1073/pnas.232591099 PMC137724

[38] D. E. Cane , R. M. Watt , Proc. Natl. Acad. Sci. U. S. A. 2003, 100, 1547–1551. 12556563 10.1073/pnas.0337625100 PMC149869

[39] P. Baer , P. Rabe , C. A. Citron , C. C. De Oliveira Mann , N. Kaufmann , M. Groll , J. S. Dickschat , ChemBioChem 2014, 15, 213–216. 24399794 10.1002/cbic.201300708

[40] V. González , D. J. Grundy , J. A. Faraldos , R. K. Allemann , Org. Biomol. Chem. 2016, 14, 7451–7454. 27431578 10.1039/c6ob01398h

[41] M. Loizzi , V. González , D. J. Miller , R. K. Allemann , ChemBioChem 2018, 19, 100–105. 29115742 10.1002/cbic.201700531 PMC5814876

[42] Y. Yoshikuni , V. J. J. Martin , T. E. Ferrin , J. D. Keasling , Chem. Biol. 2006, 13, 91–98. 16426975 10.1016/j.chembiol.2005.10.016

[43] H. Kawaide , K. I. Hayashi , R. Kawanabe , Y. Sakigi , A. Matsuo , M. Natsume , H. Nozaki , FEBS J. 2011, 278, 123–133. 21122070 10.1111/j.1742-4658.2010.07938.x

[44] P. L. Srivastava , A. M. Escorcia , F. Huynh , D. J. Miller , R. K. Allemann , M. W. Van Der Kamp , ACS Catal. 2021, 11, 1033–1041. 33614194 10.1021/acscatal.0c04647 PMC7886051

[45] M. Biasini , S. Bienert , A. Waterhouse , K. Arnold , G. Studer , T. Schmidt , F. Kiefer , T. G. Cassarino , M. Bertoni , L. Bordoli , T. Schwede , Nucl. Acids Res. 2014, 42, 252–258. 10.1093/nar/gku340 PMC4086089 24782522

[46] D. J. Tantillo , Nat. Prod. Rep. 2011, 28, 1035–1053. 21541432 10.1039/c1np00006c

[47] T. E. O'Brien , S. J. Bertolani , D. J. Tantillo , J. B. Siegel , Chem. Sci. 2016, 7, 4009–4015. 30155043 10.1039/c6sc00635c PMC6013805

[48] M. Isegawa , S. Maeda , D. J. Tantillo , K. Morokuma , Chem. Sci. 2014, 5, 1555–1560.

[49] S. R. Hare , D. J. Tantillo , Beilstein J. Org. Chem. 2016, 12, 377–390. 27340434 10.3762/bjoc.12.41 PMC4902080

[50] S. C. Kampranis , D. Ioannidis , A. Purvis , W. Mahrez , E. Ninga , N. A. Katerelos , S. Anssour , J. M. Dunwell , J. Degenhardt , A. M. Makris , P. W. Goodenough , C. B. Johnson , Plant Cell 2007, 19, 1994–2005. 17557809 10.1105/tpc.106.047779 PMC1955729

[51] Y. H. Wang , H. Xu , J. Zou , X. B. Chen , Y. Q. Zhuang , W. L. Liu , E. Celik , G. D. Chen , D. Hu , H. Gao , R. Wu , P. H. Sun , J. S. Dickschat , Nat. Catal. 2022, 5, 128–135.

[52] D. J. *Grundy, Expanding the Terpenome Complementary Approaches to Novel Terpenoids*, PhD Thesis, Cardiff University **2015**, https://orca.cardiff.ac.uk/id/eprint/88037.

[53] M. Chen , N. Al-Lami , M. Janvier , E. L. D'Antonio , J. A. Faraldos , D. E. Cane , R. K. Allemann , D. W. Christianson , Biochemistry 2013, 52, 5441–5453. 23905850 10.1021/bi400691v PMC3755762

[54] M. M. Bradford , Anal. Biochem. 1976, 72, 248–254. 942051 10.1016/0003-2697(76)90527-3

