# Supplementary material for: Single Point Mutation Abolishes Water Capture in Germacradien‐4‐ol Synthase
Source: Chembiochem. 2024 Aug 7;25(23):e202400290. doi: 10.1002/cbic.202400290 (PMC11610670; doi:10.1002/cbic.202400290)
Supplement: Supplementary file 1 — Supporting Information [file CBIC-25-e202400290-s001.pdf]

ChemBioChem

Supporting Information

Single Point Mutation Abolishes Water Capture in Germacradien-4-ol Synthase

Víctor González Requena, Prabhakar L. Srivastava, David J. Miller, and Rudolf K. Allemann*

Supporting Information

Single Point Mutation Abolishes Water Capture in Germacradien-4-ol Synthase

Víctor González Requena,[a] Prabhakar L. Srivastava,[a] David J. Miller[a] and Rudolf K. Allemann*[a]

[a]School of Chemistry, Main Building, Cardiff University, Park Place, Cardiff, CF10 3AT, United Kingdom.
E-mail: allemannrk@cardiff.ac.uk – Telephone: +44 (0)29 2068 8443.

Table of contents

**1**	Materials and general methods	**S2**
**2.**	Site direct mutagenesis (SDM).	**S3**
**3.**	Preparation of GdolS (and mutants)	**S5**
**4.**	Analytical Incubation of GdolS and mutants with FDP	**S5**
**5.**	Steady-state kinetics parameters of GdolS mutants.	**S6**
**6.**	References	**S15**

1. Materials and general methods.

Oligonucleotides and primers for site directed mutagenesis were purchased from Sigma Aldrich (UK). *Pfu* DNA polymerase and *DpnI* restriction enzyme were purchased from Fisher (UK). Site directed mutagenesis was carried out using the Quickchange site-directed mutagenesis kit (Stratagene) according to the manufacturer's instructions. QIAGEN miniprep kit was used for the purification of plasmids according to the manufacturer's instructions. All the mutated plasmids were confirmed by DNA sequence analysis using Eurofins DNA sequencing service. Protein expression was induced using isopropyl-β-D-1-thiogalactopyranoside (IPTG). An unstained protein size marker (14.4 – 116 kDa) was used to identify protein by 10% SDS-PAGE gel. An Amicon YM30 membrane was used for protein concentration. Protein concentration was measured by the Bradford method using bovine serum albumin as the calibration standard.[1] UV spectroscopy was performed using a Jasco V-660 spectrophotometer.

[1-^{3}H-FDP] was purchased from FluoroChem. Unlabelled FDP was synthesised from commercially available *trans, trans*-farnesol.[2] [1-^{3}H]-FDP was diluted by addition of unlabelled FDP to give a final specific activity of 75 mCi/mmol. All other chemicals were from Sigma-Aldrich, Fisher or Melford.

GC-MS analysis of incubation products was performed on a Hewlett Packard 6890 GC apparatus fitted with: column= J&W scientific DB-5MS column (30 m x 0.25 mm internal diameter), and a Micromass GCT Premiere detecting in the range *m/z* 50-800 in the EI$^+$ mode with scanning once a second with a scan time of 0.95 s. Method 1: injection port 100 °C; split ratio 5:1; initial pressure 1 kPa; initial temperature 80 °C (1 min hold), ramp of 4 °C/min to 180 °C (2 min hold), flow 1 mL/min; Method 2: injection port 100 °C; split ratio 5:1; initial pressure 1 kPa; initial temperature 80 °C (2 min hold), ramp of 8 °C/min to 280 °C (3 min hold), flow 1 mL/min.

2. Site direct mutagenesis (SDM).

Site directed mutagenesis was carried out using the Quickchange site directed mutagenesis kit (Stratagene) according to the manufacturer's instructions. QIAGEN miniprep kit was used for the purification of plasmids according to the manufacturer's instructions.

All the mutated plasmids were confirmed by DNA sequence analysis.

Table S1. The mutagenic primers used for preparation of GdolS variants.

Name	5' – Sequence – 3'
**GdolS – T175C**	Forward: 5' GACGCTCAGACGCGGTTGCGCCGCGATGGAGAG 3'<br>Reverse: 5' CTCTCCATCGCGGCGCAACCGCGTCTGAGCGTC 3'
**GdolS – T175D**	Forward: 5' GCTCAGACGCGGTGATGCCGCGATGGAGAG 3'<br>Reverse: 5' CTCTCCATCGCGGCATCACCGCGTCTGAGC 3'
**GdolS – T175N**	Forward: 5' CTCAGACGCGGTAATGCCGCGATGGAGAG 3'<br>Reverse: 5' CTCTCCATCGCGGCATTACCGCGTCTGAG 3'
**GdolS – A176G**	Forward: 5' CGCTCAGACGCGGTACCGGCGCGATGGAGAGCATCTTC 3'<br>Reverse: 5' GAAGATGCTCTCCATCGCGCCGGTACCGCGTCTGAGCG 3'
**GdolS – A176V**	Forward: 5' GACGCGGTACCGTGGCGATGGAGAGC 3'<br>Reverse: 5' GCTCTCCATCGCCACGGTACCGCGTC 3'
**GdolS – A176L**	Forward: 5' CTCAGACGCGGTACCCTGGCGATGGAGAGCATC 3'<br>Reverse: 5' GATGCTCTCCATCGCCAGGGTACCGCGTCTGAG 3'
**GdolS – A176I**	Forward: 5' CTCAGACGCGGTACCATTGCGATGGAGAGCATC 3'<br>Reverse: 5' GATGCTCTCCATCGCAATGGTACCGCGTCTGAG 3'
**GdolS – A176M**	Forward: 5' GACGCTCAGACGCGGTACCATGGCGATGGAGAGCATCTTC 3'<br>Reverse: 5' GAAGATGCTCTCCATCGCCATGGTACCGCGTCTGAGCGTC 3'
**GdolS – A176F**	Forward: 5' CTCAGACGCGGTACCTTTGCGATGGAGAGCATC 3'<br>Reverse: 5' GATGCTCTCCATCGCAAAGGTACCGCGTCTGAG 3'
**GdolS – A176T**	Forward: 5' CAGACGCGGTACCACCGCGATGGAGAG 3'<br>Reverse: 5' CTCTCCATCGCGGTGGTACCGCGTCTG 3'
**GdolS – A176D**	Forward: 5' CAGACGCGGTACCGATGCGATGGAGAGCATC 3'<br>Reverse: 5' GATGCTCTCCATCGCATCGGTACCGCGTCTG 3'
**GdolS – A176Q**	Forward: 5' CTCAGACGCGGTACCCAGGCGATGGAGAGCATC 3'<br>Reverse: 5' GATGCTCTCCATCGCCTGGGTACCGCGTCTGAG 3'
**GdolS – H150F**	Forward: 5' CTACTTCGCCTGCTTTCCCGCGGAGGCCG 3'<br>Reverse: 5' CGGCCTCCGCGGGAAAGCAGGCGAAGTAG 3'
**GdolS – H150Y**	Forward: 5' CTACTTCGCCTGCTACCCCGCGGAGG 3'<br>Reverse: 5' CCTCCGCGGGGTAGCAGGCGAAGTAG 3'
**GdolS – H150W**	Forward: 5' CTACTTCGCCTGCTGGCCCGCGGAGGCC 3'<br>Reverse: 5' GGCCTCCGCGGGCCAGCAGGCGAAGTAG 3'
**GdolS – H150C**	Forward: 5' CTACTTCGCCTGCTGCCCCGCGGAGGC 3'<br>Reverse: 5' GCCTCCGCGGGGCAGCAGGCGAAGTAG 3'
**GdolS – H150R**	Forward: 5' GTACTACTTCGCCTGCCGTCCCGCGGAGGCCGCCG 3'<br>Reverse: 5' CGGCGGCCTCCGCGGGACGGCAGGCGAAGTAGTAC 3'

Polymerase Chain reaction (PCR) procedure

Table S2. PCR chemical components. The reaction mixture was made up into a PCR tube according to the following table:

Component	Concentration
1. Pfu DNA polymerase buffer (5X)	1X
2. dNTPs	200 μM each
3. Fw primer	1 μM
4. Rv primer	1 μM
5. DNA template	0.1-0.2 μg/50μL
6. Primestar polymerase (2.5 U/μL)	1.25 U
7. Nuclease-free water to make up the final volume of	50 μL

Table S3. PCR protocol. PCR was carried out in a thermocycler using the following protocol:

Step		Temperature (°C)	Time (min)
**Initial denaturation**		95	3
**Denaturation**		95	1
**Annealing**	**x 33**	55-62	2
**Extension**		72	12
**Final extension**		72	10

The PCR mixture was digested with 0.5 μL of *Dpn*I (10 U/μL) for 1 hour at 37 °C to cleave the parental DNA template. Then, the mixture was cooled on ice and transformed into XL1-blue *E. coli* cells, which were prepared according to manufacturer instructions.

Mutations were confirmed by DNA sequencing using Eurofins MWG Operon's DNA sequencing service.

3. Preparation of GdolS (and mutants).

GdolS and all the variants were expressed in *E. coli* BL21(DE3) cells and purified using Ni-NTA affinity column chromatography to the homogeneity and used for enzymatic characterisation.

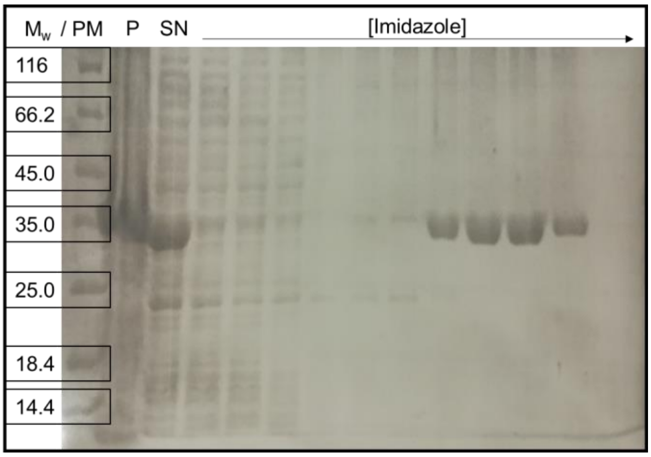

Figure S1. 10% SDS-polyacrylamide gel of GdolS. PM: protein marker; P: pellet; SN: supernatant and, [imidazole]: imidazole gradient.

4. Analytical Incubation of GdolS and mutants with FDP.

A solution of 1 µM GdolS or GdolS variants and 200 µM FDP in incubation buffer [250 mL, 50 mM Tris, 5 mM βME, 5 mM $MgCl_2$, pH 8.0] was prepared. The aqueous layer was overlaid with HPLC grade pentane (0.5 mL) and the resulting mixture was incubated (8 - 18 h) at 25 °C. The incubations were repeated without enzyme as negative controls. Samples were vortexed and pentane extracts were then analysed by gas chromatography-mass spectrometry (GC-MS) according to General Methods (Section 1). All the incubations were performed in triplicates. Peak areas were calculated for all the products observed from GdolS as well as all the variants. The observed peak areas are then used to generate the product ratios for each variant.

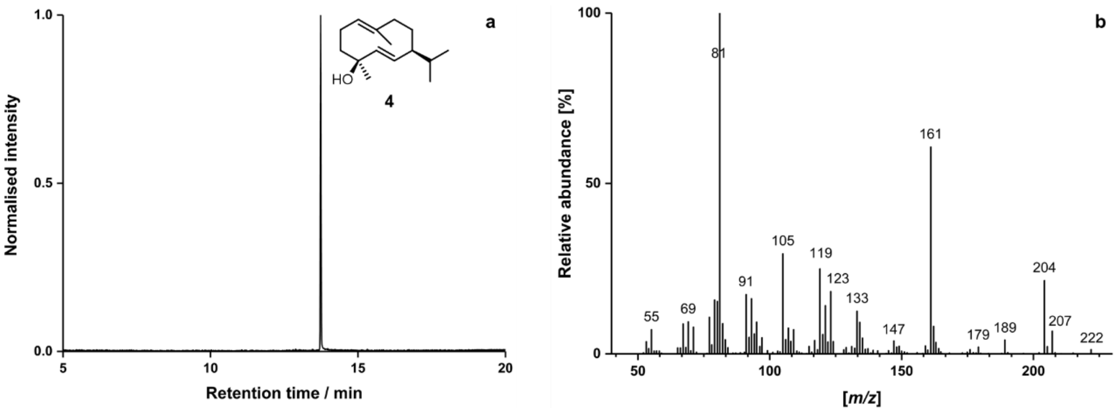

Figure S2. a) Total ion chromatogram of the pentane extractable products arising from the incubation of GdolS with FDP (**1**), method 2. **b)** Mass spectrum of (-)-germacradien-4-ol (**4**).

5. Steady-state kinetics parameters of GdolS mutants.

Kinetics assays were carried out according to the standard, linear range, micro-assay procedure previously developed for GdolS.[3] Reactions (final volume of 250 μL) were initiated by the addition of enzyme (100 nM) to assay buffer solutions [50 mM HEPES, 2.5 mM $MgCl_2$, and 5 mM 2-mercaptoethanol (pH 8.0)] containing 0−20 μM [1-^{3}H]-FDP and overlaid with 1 mL of hexane. After incubation for 10 min, reactions were quenched by addition of EDTA (50 μL, 0.5 M) and vortexed for 30 s. The hexane layer was separated, and the sample extracted with hexane and Et_2O (11:1) in the same way (2 × 750 μL). The pooled organic extracts were passed through a short column of silica (~500 mg) into 15 mL of Ecoscint fluid (National Diagnostics), and the silica was then washed with a further portion of hexane and Et_2O (750 μL) and radioactive mixture was analysed using a TRI-CARB 2900TR Liquid Scintillation Analyzer.

Steady-State kinetic parameters for GdolS mutants were obtained by direct fitting of the data to the Michaelis-Menten equation by nonlinear least squares regression in conjunction with the graphical procedures developed by Lineweaver-Burk using the commercial SigmaPlot package (Systat Software).

The k_{cat} and K_M values were determined to be 0.068 ± 0.001 s^{-1} and 1.02 ± 0.09 μM respectively, which are in good agreement with those reported previously in our laboratory (0.079 ± 0.003 s^{-1} and 1.07 ± 0.13 μM).[3]

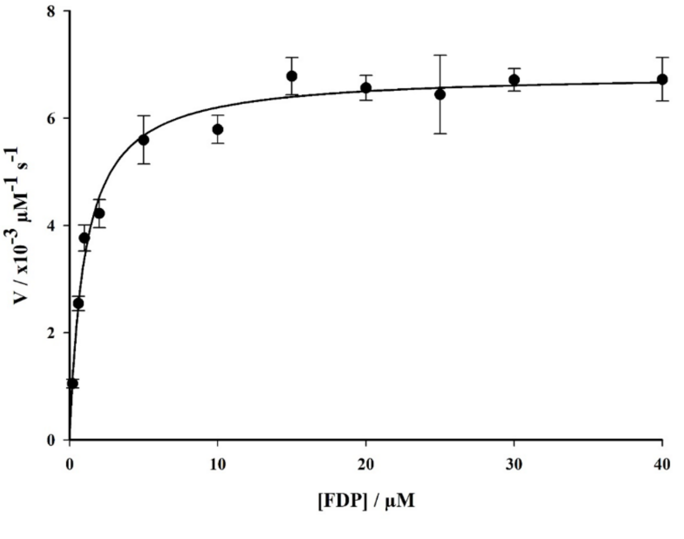

Figure S3. Representative Michaelis-Menten graph of steady-state kinetic parameters of GdolS.

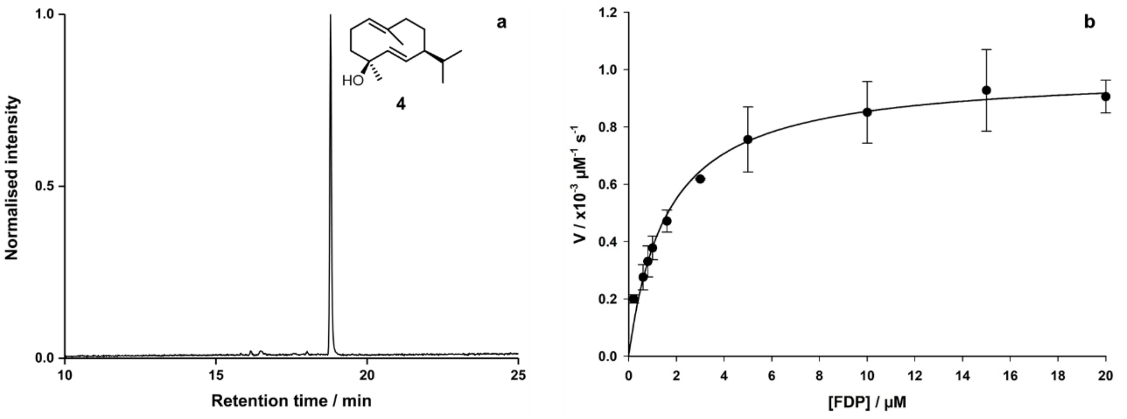

Figure S4. **a)** Total ion chromatogram of the pentane extractable products arising from incubation of FDP (**1**) with GdolS-A176G, method 1. **b)** Michaelis-Menten graph for the calculation of steady-state kinetic parameters of GdolS-A176G.

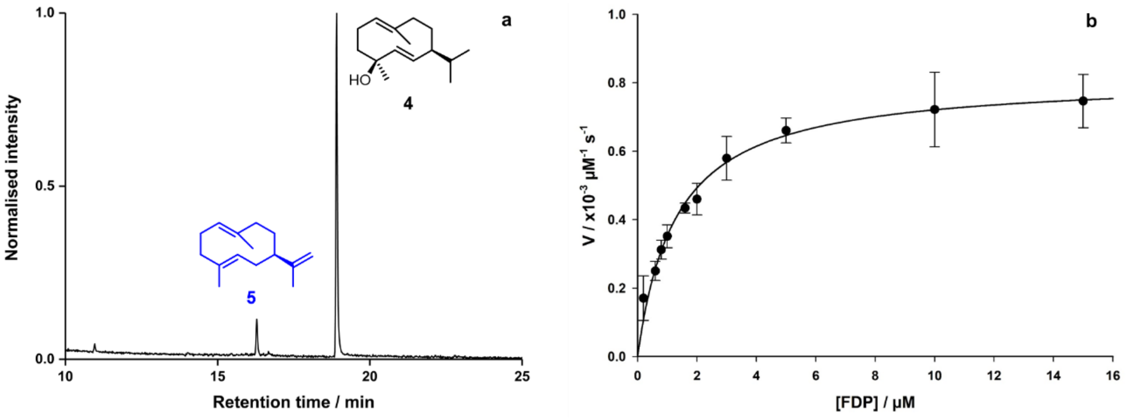

Figure S5. a) Total ion chromatogram of the pentane extractable products arising from incubation of FDP (**1**) with GdolS-A176V, method 1. **b)** Michaelis-Menten graph for the calculation of steady-state kinetic parameters of GdolS-A176V.

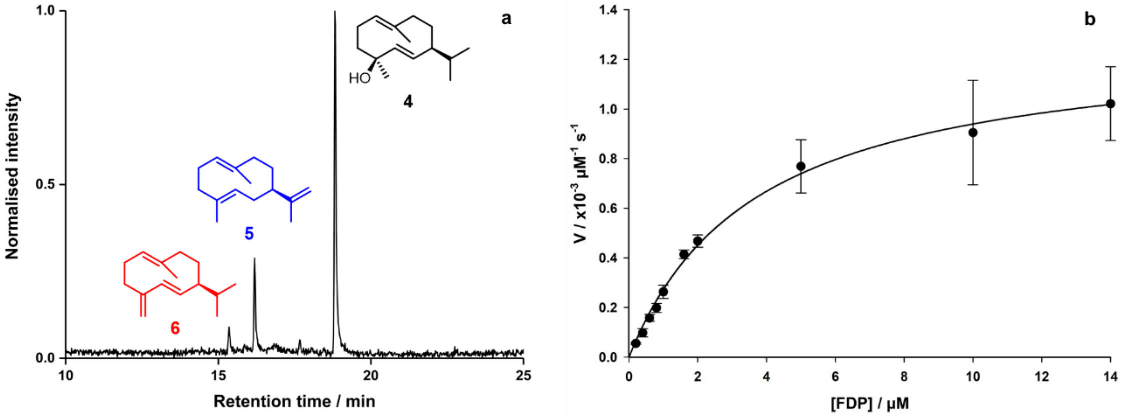

Figure S6. a) Total ion chromatogram of the pentane extractable products arising from incubation of FDP (**1**) with GdolS-A176L, method 1. **b)** Michaelis-Menten graph for the calculation of steady-state kinetic parameters of GdolS-A176L.

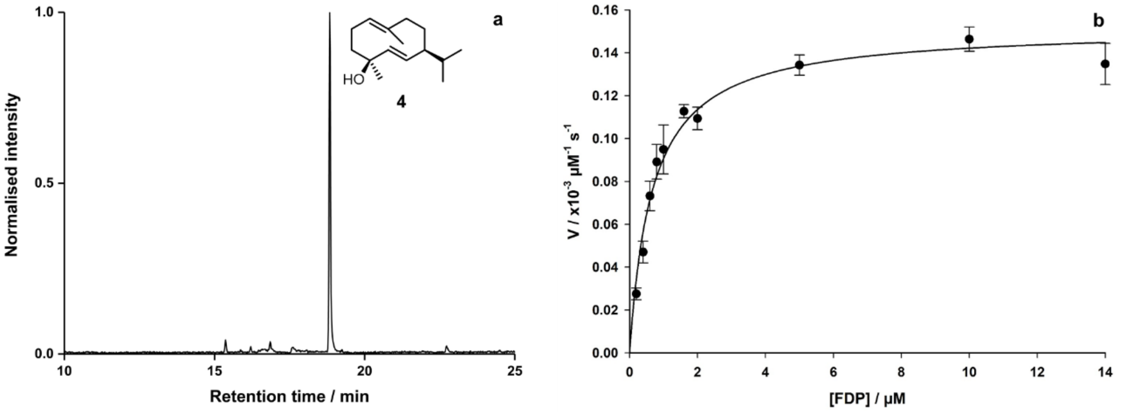

Figure S7. a) Total ion chromatogram of the pentane extractable products arising from incubation of FDP (**1**) with GdolS-A176T, method 1. **b)** Michaelis-Menten graph for the calculation of steady-state kinetic parameters of GdolS-A176T.

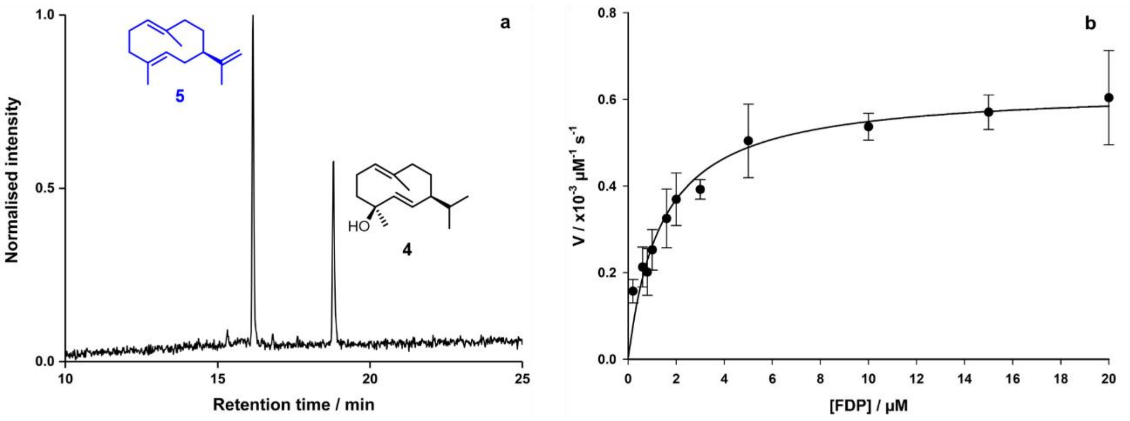

Figure S8. a) Total ion chromatogram of the pentane extractable products arising from incubation of FDP (**1**) with GdolS-A176I, method 1. **b)** Michaelis-Menten graph for the calculation of steady-state kinetic parameters of GdolS-A176I.

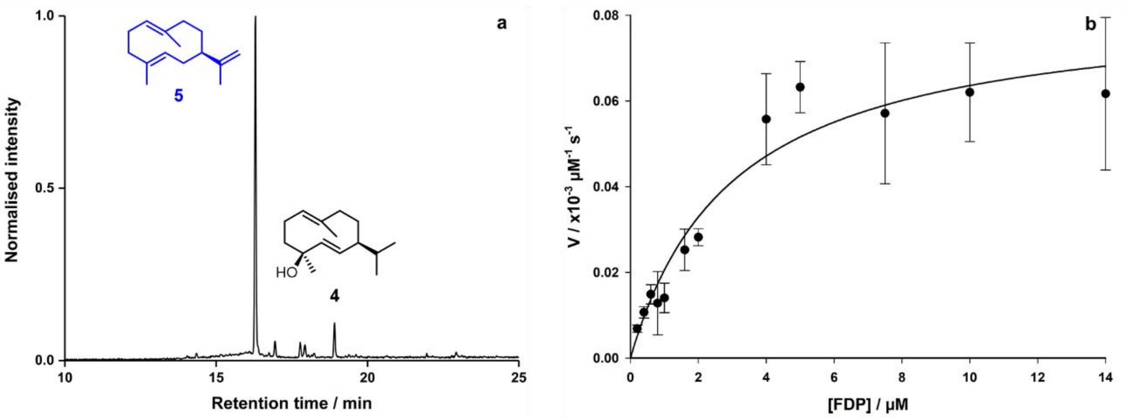

Figure S9. a) Total ion chromatogram of the pentane extractable products arising from incubation of FDP (**1**) with GdolS-A176M, method 1. **b)** Michaelis-Menten graph for the calculation of steady-state kinetic parameters of GdolS-A176M.

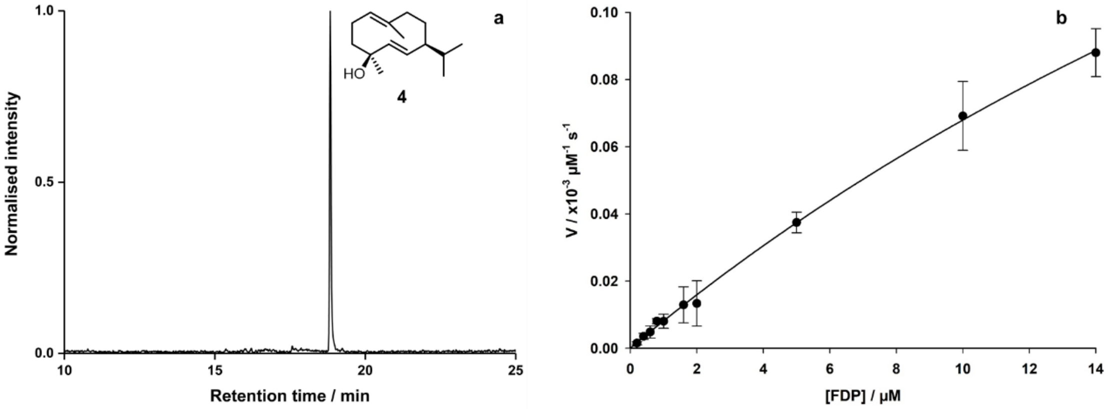

Figure S10. a) Total ion chromatogram of the pentane extractable products arising from incubation of FDP (**1**) with GdolS-A176Q, method 1. **b)** Michaelis-Menten graph for the calculation of steady-state kinetic parameters of GdolS-A176Q.

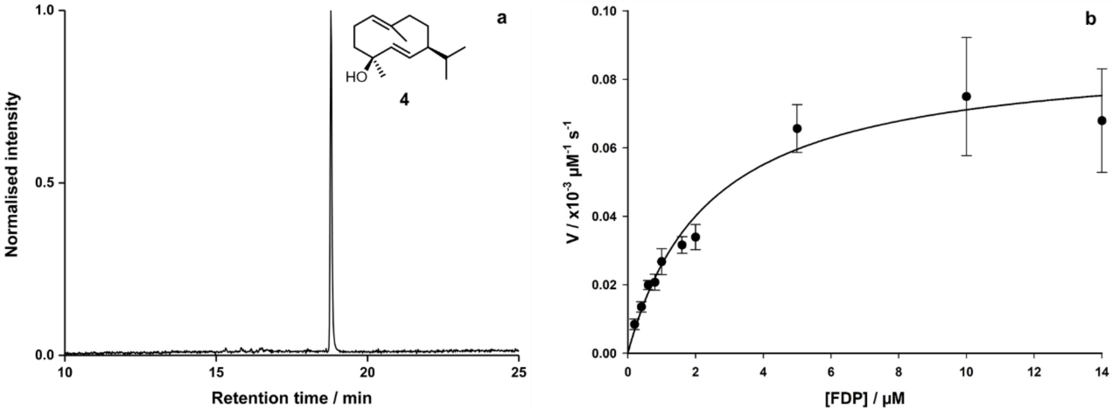

Figure S11. a) Total ion chromatogram of the pentane extractable products arising from incubation of FDP (**1**) with GdolS-A176D, method 1. **b)** Michaelis-Menten graph for the calculation of steady-state kinetic parameters of GdolS-A176D.

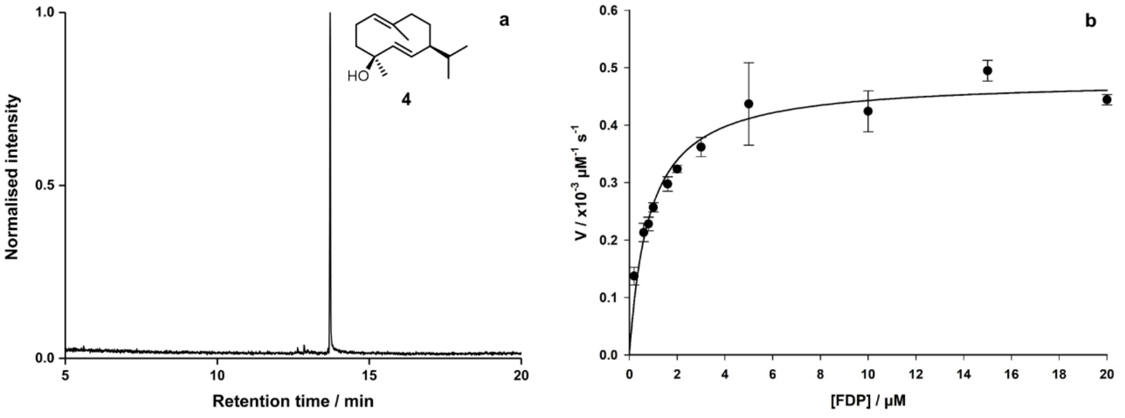

Figure S12. a) Total ion chromatogram of the pentane extractable products arising from incubation of FDP (**1**) with GdolS-T175C, method 2. **b)** Michaelis-Menten graph for the calculation of steady-state kinetic parameters of GdolS-T175C.

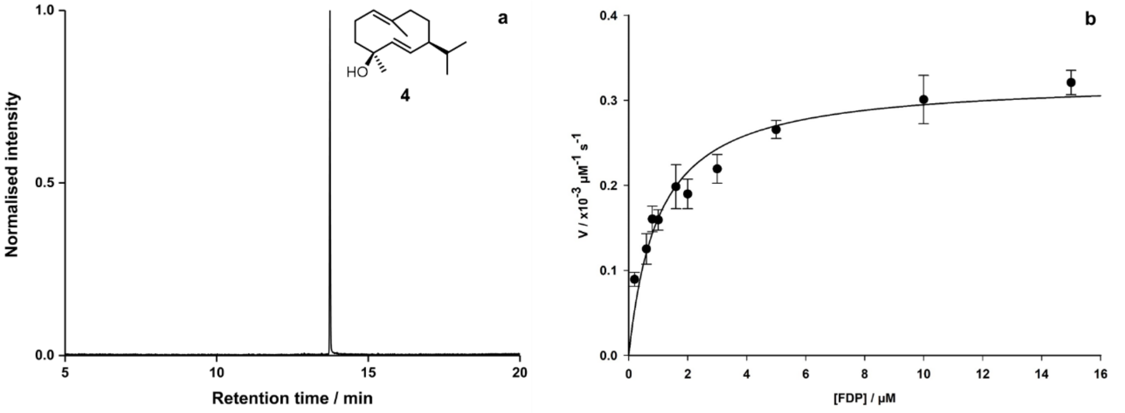

Figure S13. a) Total ion chromatogram of the pentane extractable products arising from incubation of FDP (**1**) with GdolS-T175N, method 2. **b)** Michaelis-Menten graph for the calculation of steady-state kinetic parameters of GdolS-T175N.

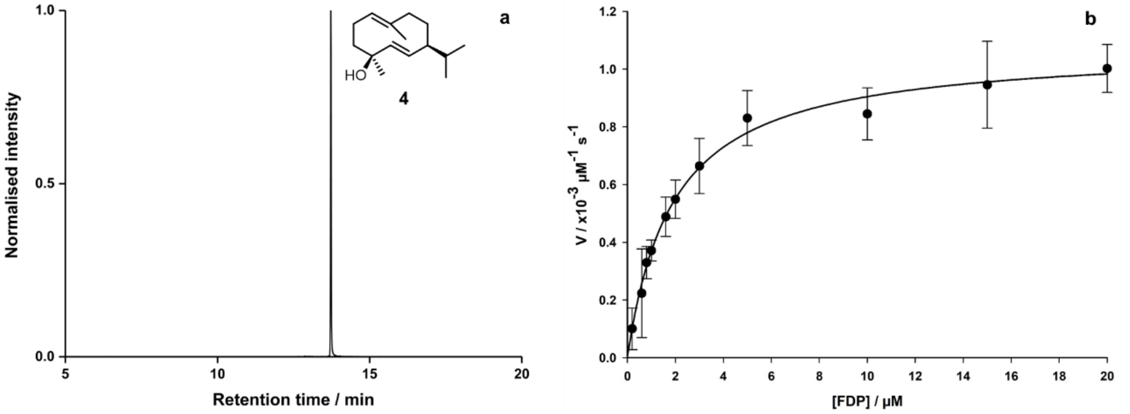

Figure S14. a) Total ion chromatogram of the pentane extractable products arising from incubation of FDP (**1**) with GdolS-H150Y, method 2. **b)** Michaelis-Menten graph for the calculation of steady-state kinetic parameters of GdolS-H150Y.

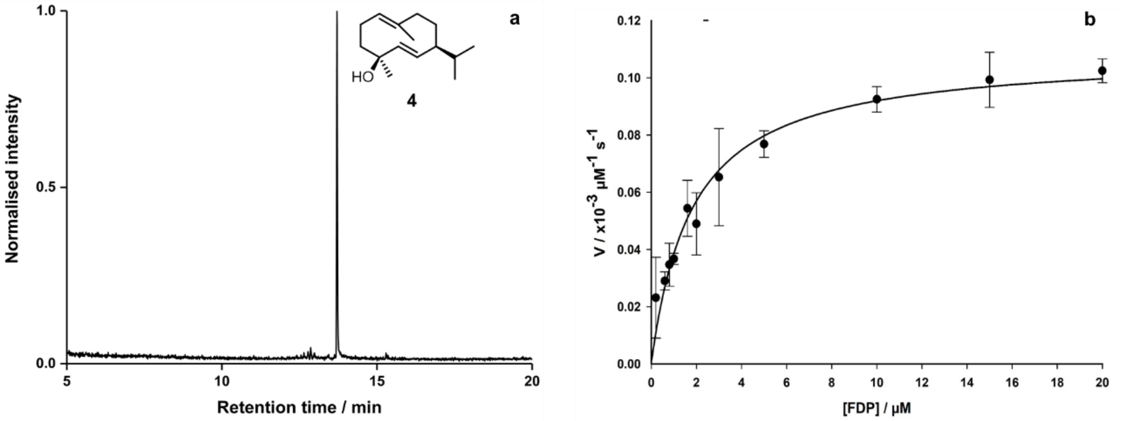

Figure S15. a) Total ion chromatogram of the pentane extractable products arising from incubation of FDP (**1**) with GdolS-H150F, method 2. **b)** Michaelis-Menten graph for the calculation of steady-state kinetic parameters of GdolS-H150F.

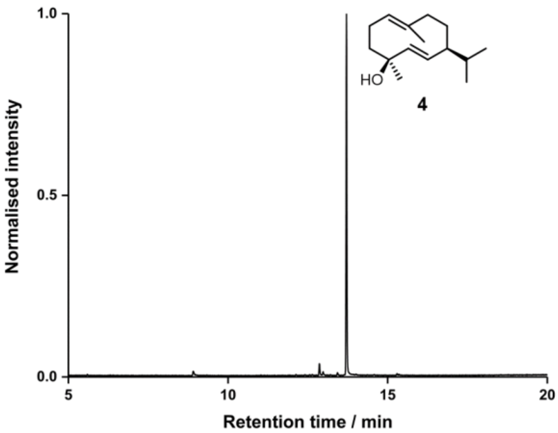

Figure S16. a) Total ion chromatogram of the pentane extractable products arising from incubation of FDP (**1**) with GdolS-H150C, method 2.

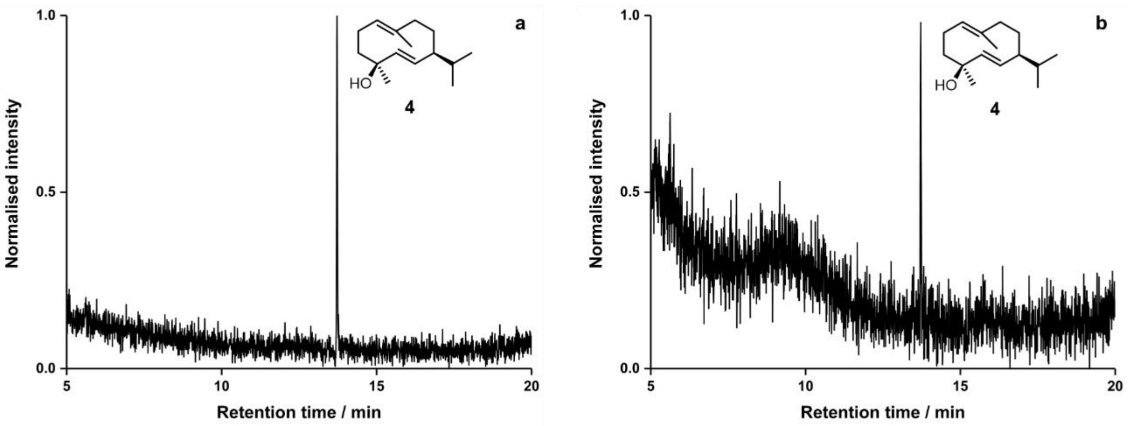

Figure S17. **a)** Total ion chromatogram of the pentane extractable products arising from incubation of FDP (**1**) with GdolS-H150W, method 2. **b)** Total ion chromatogram of the pentane extractable products arising from incubation of FDP (**1**) with GdolS-H150R, method 2.

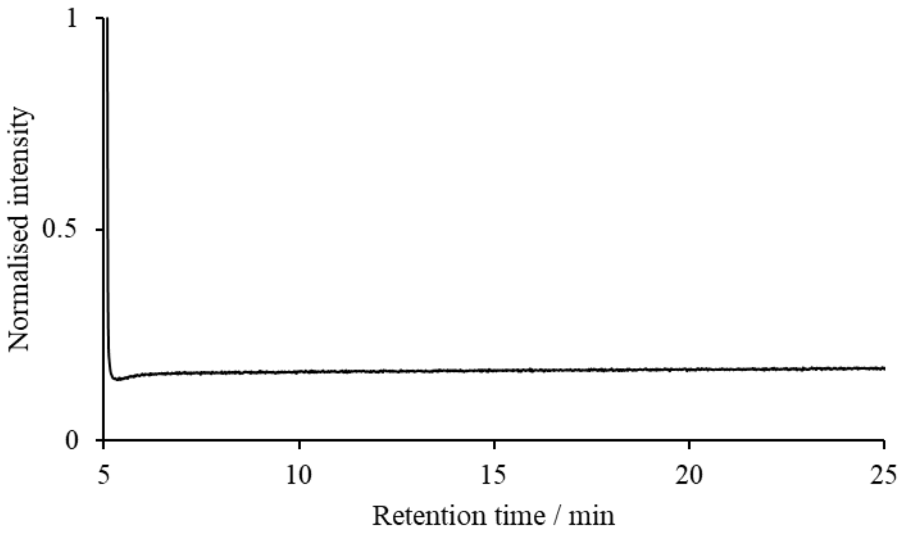

Figure S18. Total ion chromatogram of the pentane extractable products arising from incubation of FDP (**1**) with GdolS-A176F, method 2.

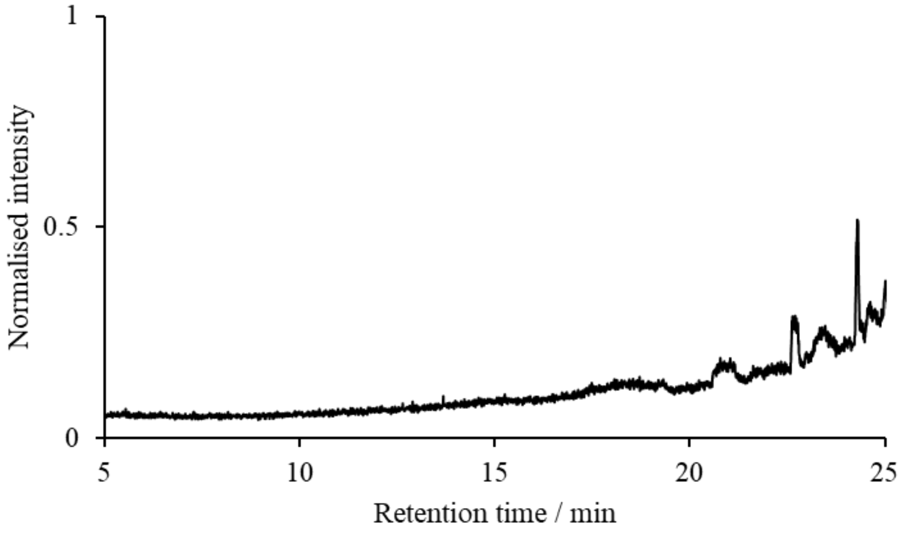

Figure S19. Total ion chromatogram of the pentane extractable products arising from incubation of FDP (**1**) with GdolS-T175D, method 2.

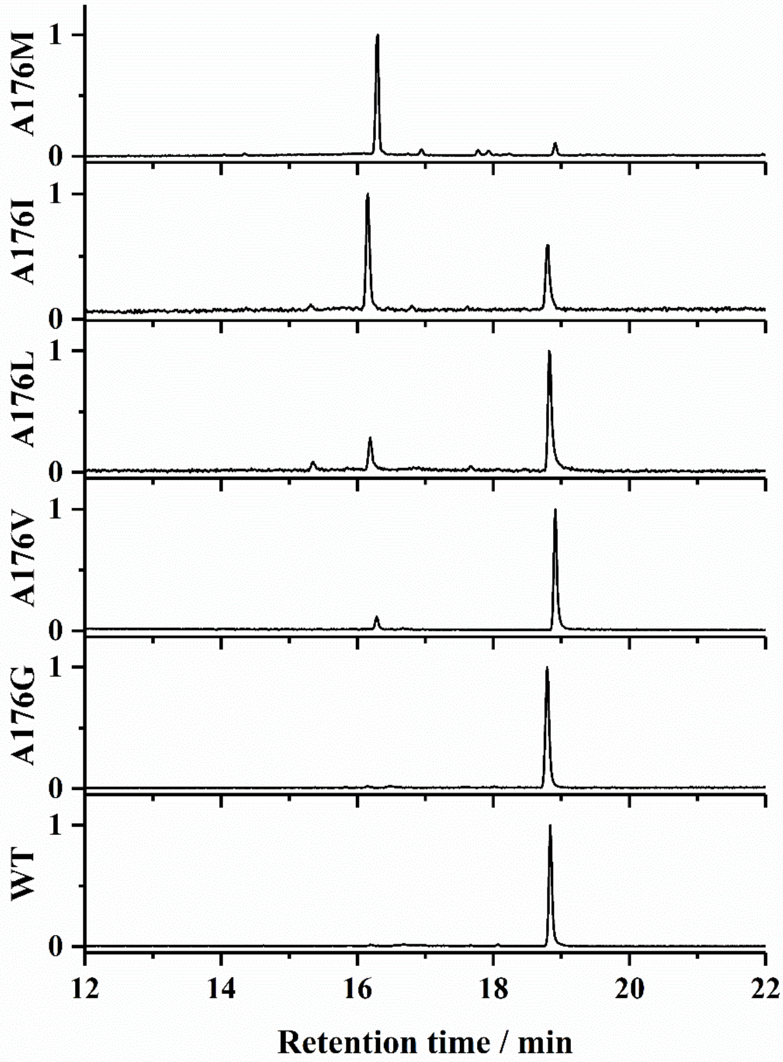

Figure S20. Total ion chromatograms of the pentane extractable products arising from incubation of FDP (**1**) with GdolS and A176 non-polar mutants, method 1.

6. References:

1) M. M. Bradford, *Anal. Biochem.* **1976**, *72*, 248–254.
2) V. J. Davisson, A. B. Woodside, T. R. Neal, K. E. Stremler, M. Muehlbacher, C. D. Poulter, *J. Org. Chem.* **1986**, *51*, 4768–4779.
3) D. J. Grundy, M. Chen, V. González, S. Leoni, D. J. Miller, D. W. Christianson, R. K. Allemann, *Biochemistry* **2016**, *55*, 2112–2121.
